# Balancing Acts: Exploring Well‐Being in Sandwiched and Non‐Sandwiched Caregivers

**DOI:** 10.1002/alz70858_097629

**Published:** 2025-12-24

**Authors:** Virginia T Gallagher, Natasha L Nemmers, Yuchen Zhang, Sarah E. Patterson

**Affiliations:** ^1^ University of Virginia, Charlottesville, VA, USA; ^2^ Wayne State University, Detroit, MI, USA; ^3^ University of Pittsburgh, Pittsburgh, PA, USA; ^4^ University of Michigan, Ann Arbor, MI, USA

## Abstract

**Background:**

The population caring for both an older adult and a minor child, known as “sandwiched” caregivers, is growing due to demographic shifts. Most research on the sandwiched caregivers exclusively focuses on adult children, overlooking others like neighbors or grandchildren. Moreover, little is known about sandwiched caregivers of persons living with dementia. This study compares well‐being of sandwiched and non‐sandwiched caregivers of individuals with and without dementia, with a focus on the role of additional help from family and friends and restriction in social activities.

**Method:**

Data from 1,728 caregivers of 1,156 older adults were analyzed from the 2022 National Health and Aging Trends Study (NHATS) and National Study of Caregiving (NSOC). Four groups were compared: Sandwiched, Dementia (S+D); Sandwiched, No Dementia (S‐D); Non‐Sandwiched, Dementia (NS+D); Non‐Sandwiched, No Dementia (NS‐D). Linear regressions predicted caregiver well‐being, adjusting for demographic and care‐related variables, with social participation restriction and family/friend help as moderators. Analyses are weighted and adjusted for NHATS/NSOC's complex survey design.

**Results:**

A majority of S+D caregivers were biological, step, or in‐law children of the person with dementia (72%), followed by other relatives (21%), and unpaid caregivers like friends or neighbors (7%). Sandwiched caregivers are more likely to receive additional help, be employed, and belong to racial‐ethnic minority groups relative to non‐sandwiched caregivers (*p*s< .001). In terms of well‐being, compared to S+D caregivers, S‐D caregivers had significantly higher well‐being (*B* = 2.91, *p* < .001), as did NS+D caregivers (*B* = 1.66, *p* = .021), but not NS‐D caregivers (*B* = 1.03, *p* = 163). Linear regression interaction effects revealed differential impacts of social restriction on well‐being across caregiver groups, such that S‐D caregivers (*B* = ‐1.73, *p* < .001) and NS+D (*B* = ‐1.01, *p* = .013) report significantly worse well‐being associated with increased participation restriction compared to S+D. Additional support from family and friends did not significantly moderate the relationship between caregiver groups and well‐being.

**Conclusion:**

This research underscores vulnerability in well‐being among sandwiched caregivers, especially those involved in dementia care. While sandwiched caregivers often have additional care support, those caring for non‐dementia care recipients are particularly sensitive to restrictions in social activities due to caregiving. Interventions are needed to address the wellbeing needs of sandwiched caregivers.

